# Identification of social innovation in health criteria in Latin America

**DOI:** 10.1136/bmjopen-2022-063205

**Published:** 2022-06-09

**Authors:** Luisa Fernanda Duque-Paz, Diana Castro-Arroyave

**Affiliations:** 1 Secretaría de Integración Social, Alcaldía de Palmira, Palmira, Colombia; 2 Centro Internacional de Entrenamiento e Investigaciones Medicas -CIDEIM-, SIHI LAC Hub, Universidad Icesi, Cali, Colombia

**Keywords:** public health, social medicine, infectious diseases, preventive medicine, qualitative research

## Abstract

**Objectives:**

Understanding the Latin American Social Innovation in Health (SIH) approach requires a process of typifying and identifying main criteria of the approach based on the employed practices of different health initiatives implemented throughout the region. This article presents a descriptive analysis of the main criteria of SIH.

**Design:**

To identify the theoretical and methodological developments of SIH between the years 2013 and 2018, a scoping review was conducted using a mixed approach. 80 texts in English, Spanish and Portuguese were screened through a reflexive analysis process involving intratextual and intertextual reading.

**Setting and participants:**

The documentary research covered journals, books and higher degree theses addressing experiences or theoretical constructs developed in the Latin American region.

**Primary and secondary outcome measures:**

The approaches identified in the studied initiatives were mutually complementary; moreover, based on the typification of the main criteria between approaches and implementation proposals, the convergences and divergences between SIH and other approaches found in the sample were identified. In most cases, the different approaches in the sample are committed to initiatives that include some degree of innovation, improve access to healthcare services and recognise in one way or another a public policy in line with the Sustainable Development Goals (SDGs).

**Results:**

Eighteen characteristic criteria were identified, of which nine particularly differentiate SIH from other approaches conceptually and methodologically. Further work is essential to eliminate the vague delimitation between social and technological aspects of innovation.

**Conclusions:**

The findings indicate that although the SIH concept is in construction, it is advancing down a path of recognition in the region, defining its role as an important field of study on social transformation in health and development.

**Strengths and limitations of this study**

- The key strength of this study is that it identified recurrent Social Innovation in Health (SIH) criteria from different Latin American initiatives.
- The identification of these 18 criteria contributes to advance towards the conceptual and methodological delimitation of SIH as a process or an approach.
- The results facilitate the identification of SIH initiatives based on key criteria that favour the creation of a frame of reference.
- Although the review was based in the Latin American region context, the identified criteria is prone to have wider applicability.

## Background

Health and well-being as a permanent condition have always been a challenging ideal among low-income and middle-income countries, where inequality, social vulnerability, deficient state involvement, a lack of public policies and neglected diseases of poverty are constant difficulties. Considering that areas located in the Latin American countryside are generally the poorest and less-favoured regions,^1^ access to healthcare services is a dilemma, a challenge and a persistent need to join efforts to deliver efficient solutions and guarantee universal health access and service quality exists.

Throughout the region, health systems tend to be weak, fragmented, geographically centralised, with specialty services such as specialised diagnostics and medical consultations concentrated in major cities, receiving considerably low investments of about an average of 3.5% of the countries’ Gross Domestic Product.^2^ In Latin America, this implies great challenges to achieving universal health access and aligning with governmental and non-governmental efforts related to the Sustainable Development Goals (SDGs), particularly with goal three ‘Health and Well-being’, so it is essential to propose interdisciplinary and intersectoral participatory strategies to identify, differentiate and typify various and scattered efforts from different initiatives in health.

Aiming to identify, strengthen and connect the creation of solutions to local health problems focused on social transformation in health, the Social Innovation in Health Initiative (SIHI) Global programme led by TDR (UNDP/World Bank/WHO Special Programme for Research and Training in Tropical Diseases)/WHO started in 2014, and arrived as a programme in Latin America in 2017 with CIDEIM (Centro Internacional de Entrenamiento e Investigaciones Médicas) assuming the leading role. Since then, SIH has positioned itself as an emerging concept and as an approach that is still under construction, is defined as a participatory process that proposes and implements novel ideas that turn the SDGs into concrete actions and achievements in communities in order to solve social problems in health and generate added value to society.^3^

Acknowledging that the path of SIH in Latin America is an ongoing process, this review identified that existing approaches to health have emerged to address the health-related needs and problems that afflict populations. Approaches such as innovation in health and technological innovation in health, among others, have not yet been fully developed conceptually.

With the interest of promoting the approach in Latin America, a literature review was conducted to build a baseline of local advances in health that were consistent with the SIH approach. The screening, under the scoping review modality, selected 80 texts published between 2013 and 2018 in English, Spanish and Portuguese, and allowed to both advance in the understanding and conceptual scope of the SIH approach^4^ as well as recognise approaches and related criteria that can act as references for the identification of SIHI and guidelines for the development of new solutions based on this perspective.

This scoping review yielded insights that indicate considerable future research is still necessary to advance in the conceptual and methodological delimitation of the SIH approach. Furthermore, this article summarises the most characteristic criteria of the SIH approach, based on a comprehensive reading of different health initiatives applied throughout the region to identify conceptual and methodological advances.

## Methods

This scoping review was based on the Preferred Reporting Items for Systematic Reviews and Meta-Analyses-extension for Scoping Reviews Checklist,^5^ which was modified by the researchers to identify the Latin-American adaptation of the SIH approach and the criteria that characterise it. The implemented methodology involved both quantitative and qualitative strategies^6^ in all stages, from the search through the analysis, in which an intra and inter- textual reading approach was implemented. For a better understanding and an organised research structure, the process was developed in three stages and five steps.

### Stage 1: delimitation and criteria definition

#### Step 1: parameterisation for search term selection

With the help of the thesauri of the United Nations Educational, Scientific and Cultural Organisation (UNESCO), the United Nations Bibliographic Information System and the macrothesaurus of the Organisation for Economic Cooperation and Development, relevant descriptors and related terms were delimited to guide the search of the documentary material in order to guarantee the effectiveness and quality of retrieved information. Descriptors and related terms were selected based on their relationship with the conceptual categories, ‘social innovation’ (SI) and ‘health’, in English, Spanish and Portuguese. The selected descriptors were: innovations, social change, development, social development, technology transfer, social service, development projects, development programmes, social programme, social policy and health.

The most related descriptors of SI emerged as social change, social development, social service, development projects and social policy. To carry out the search, these descriptors and their related concepts were added to the descriptor ‘health’, using the five Boolean operators ‘and’, ‘or’ and ‘not’.

#### Step 2: selection of online databases and institutional repositories

The information sources used were Google Scholar and Scholarly, also, well known databases as PubMed, Scopus, Web of Science, Scielo and reputable Latin American research databases such as LILACS and REDALYC, which are specialised in open access and scientific and technical documentary material on health and multidisciplinary studies. Institutional repositories of public and private Colombian universities were also used.

#### Step 3: inclusion and exclusion criteria

According to the research objective, articles, books, book chapters, undergraduate and graduate theses and project reports in English, Portuguese and Spanish that present case studies of initiatives that originated and took place in Latin America between 2013 and 2018 were included, considering that in 2018 the SIHI LAC Hub began its work in the region.

Exclusion criteria consisted of documentary material on health initiatives linked to entrepreneurship or social entrepreneurship, case studies that did not take place in Latin America, languages different than the ones defined, and documents produced in a different time period than this search. Additionally, four conceptual criteria elaborated by SIHI in 2017 for their SIHI calls were considered: Innovation degree, inclusion, efficacy and affordability.

### Stage 2: documentary material search and selection

A series of Excel matrices and the software Endnote (Clarivate Analytics, Philadelphia, USA) were designed and used for the recording of the documentary material found, simplifying data analysis.

#### Step 4: online database and institutional repository search and selection

An initial search using the descriptors ‘SIH’, ‘Innovación Social en Salud’, ‘Inovação Social em Saúde’ did not retrieve sufficient information but neither did yield any conceptual difference between the categories in the three languages. A second search was performed based on the related concepts ‘Innovation’, ‘SI’ and ‘Health’ for a more efficient and effective retrieval of fitting samples.

After registering the search results, a screening was applied by verifying the thematic suitability by carefully reading each abstract, introduction, conclusions and key words to select the material according with the inclusion criteria, the first outcome left approximately 190 texts and the second and final an 80-text sample.

### Stage 3: quantitative and qualitative analysis of the data

To achieve the main objective, an intratextual and intertextual reading of the 80-text sample was performed. In this stage, a descriptive analysis using a complementary approach established the following categories of analysis: (1) Conceptual development, (2) Conceptual approach, (3) Methodological approach, (4) Success criteria, (5) Innovation degree, (6) intended purpose, (7) Implemented SIH criteria and (8) Health scope.

#### Step 5: intratextual and intertextual reading

An intratextual reading and analysis of each text was carried out to classify the texts to categories according to their content. An intertextual analysis was also carried out to identify the relationships and differences between the authors’ approaches to SIH, as well as the conceptual and methodological gaps. The triangulation of information extracted from the 80 texts facilitated the identification of the criteria referring to SIH as an approach to solve local health problems.

### Patient and public involvement

Patients and the public were not involved in the design, conduct, reporting or dissemination plans of this research.

## Results

This scoping review of the 80-text sample distributed in 38 publications in English, 37 in Spanish and 5 in Portuguese enabled the analysis of the methodological and conceptual developments of SIH, a better description of these results can be found in a more comprehensive way in Castro-Arroyave and Duque-Paz.^4^

This study will focus on specifying and identifying the attributable criteria for SIH as well as discussing the contributions of the SIHI programme and the findings of this analysis.

### Contributions of SI criteria to SIH

Some authors have spoken of criteria representing key characteristics of SI such as being eligible for policy intervention, being clearly distinguishable from other types of innovation,^7^ meeting social needs, creating new social relationships, benefiting society and improving its capacity to act,^8^ promoting human development, transforming social relations,^9^ change practices or social structure, contribute to urban and community development, improve work forms and processes, imbue technological innovations with cultural meaning and relevance, resignify social work and innovate through digital connectivity;^10^ as evidenced, the specific case of ‘SIH’ has been seldom addressed, compared with ‘Innovation’ and ‘SI’.

Nova Terra, a training programme for young people ascribed to the Nova centre per la innovació social, is a Catalan institution that promotes movement and organisation consultants for social change,^11^ recognises additional criteria for understanding SI such as, integral sustainability, deliberative participation, interactive communication, resource sharing, inclusive globalism, intercultural pluralism and gender and generational balance. Criteria compatible with those proposed in North, Central and South America.

Another Economic Commission for Latin America and the Caribbean (ECLAC) document^12^ set out a series of criteria referring to key elements for identifying an initiative as innovative. The authors organise the criteria into three processes: application, evaluation and awarding.

SI has an approximately 20-year journey, its approach has a variety of contributions,^13 14^ and it has been put to service in different disciplinary fields, such as in health. SIH emerged in 2014 through the global initiative, SIHI, whom used the criteria set that the ECLAC^15^ conceives as necessary for the identification of innovative initiatives in health as reference: effectiveness, inclusion, and degree of innovation and affordability. These criteria have been enriched by different hubs that operate in other regions of the world by autonomously including their own, and adapting others based on their own context, health programmes and research interests.^16^

In 2019, CIDEIM, the SIHI-LAC Hub leader, updated the criteria for the identification of SI in the region that aim to improve access to healthcare, and strengthen the quality of health services and diagnosis, as well as the treatment and prevention of infectious diseases; under the relevant selection criteria, degree of innovation, inclusion, affordability, effectiveness, scalability, sustainability, participation and changes in the system were applied in the crowdsourcing process carried out to identify SIHI.^17^

The aforementioned contributions converge in at least 11 criteria: sustainability, inclusion, meeting social needs, creating new or transforming social relationships, empowerment, participation, promoting human development, degree of innovation, affordability, scalability and sustainability. These criteria help to define what SI and SIH are both conceptually and methodologically, criteria that were the result of a collaborative effort among leaders of the SIHI-Hubs located in Malawi, Uganda, Philippines and Colombia.

Eighteen key criteria were identified to facilitate the comprehension and delimitation of SIH as a result of this scoping review. Table 1 presents the 18 criteria identified in this review, of which 9 are shared with or taken from SI, and another 9 specifically attributed to the SIH approach.

**Table 1:** Key criteria for SIH

Criteria shared with SI	1	Active participation of civil society
	2	Seeks sustainability
	3	Promotes resourcefulness
	4	Seeks horizontalisation of power relations with a gender perspective
	5	Introduces new ideas
	6	Promotes human progress in social environments
	7	Adheres to social development policies such as the Sustainable Development Goals, healthy cities, among others
	8	Considers the use of technological applications and technological developments
	9	Looks for scalability and replicability
SIH distinctive criteria	10	Focuses on health promotion
	11	Improves access to healthcare services
	12	Local level development with socioecological perspectives on health
	13	Implements health education approach strategies
	14	Emphasises scientific evidence
	15	Promotes interdisciplinarity and intersectorality
	16	Aligns with the model of the social determinants of health
	17	Addresses specific health needs and has an additional impact on other collective problems
	18	Adheres to health normative frameworks such as the Declaration of Alma Ata and the Ottawa Charter

SI, social innovation; SIH, Social Innovation in Health.

### Approaches found in the sample

The analysis process yielded 19 approaches that were compatible with SIH, given their affinity in objectives, methodological processes, criteria; among others shared aspects. Table 2 shows the approaches organised in three ranges of compatibility with SIH by percentage, which were estimated by calculating the number of shared criteria over the total number of criteria.

**Table 2:** Approaches grouped by compatibility range

Groups by compatibility range	Approach	Compatibility with SIH
I 67%–99%	Ecohealth	94%
	Social Innovation	83%
	Sustainable and Sanitary Territories	83%
	Psychosocial approach	78%
	Sustainable development and health promotion	72%
	Environmental health	67%
II 33%–66%	Ecosystem management	61%
	Eco-bio-social	50%
	Health Education (includes Social Pedagogy-Social Appropriation of Knowledge)	50%
	Technologies for social inclusion	44%
	Social innovation in public health	39%
	e-Public health focused on equity	33%
III 0%–32%	Scholar scientific inquiry (SI)	28%
	Care in the territory	22%
	Innovation in health	22%
	Technological Innovation in health	22%
	Disruptive innovation for healthcare service delivery	22%
	Technological transfer	17%
	Local innovative and productive system approach	0%

SIH, Social Innovation in Health.

The rank one group consists of six approaches with a range compatibility of 67%–94%, the four approaches with greater proximity to SIH are Ecohealth, SI, Psychosocial approach and Sustainable and Sanitary Territories (in Spanish, Territorios Saludables y Equitativos also referred by some authors as Healthy Cities). These approaches address criteria that articulate sustainability, equity, health promotion, disease prevention and social inclusion strategies that aim to improve the quality of life from an integral and transformative vision of health.

### Relationship between criteria and the approaches

**Figure 1:**
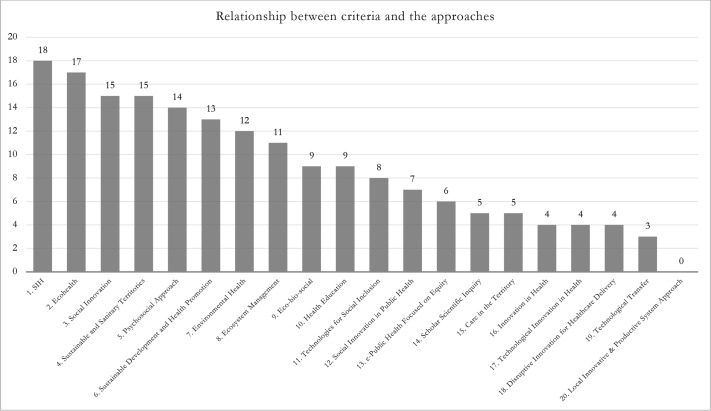
Relationship between criteria and the approaches.

Subsequently, a relationship between criteria and the approaches was established to identify the number of criteria that are considered by each different approach (see figure 1).

The 18 criteria underwent a quantitative analysis process to define which were the most consistent or recurrent within the texts. This process showed that 13 criteria were present in at least 4 of the 6 approaches corresponding to rank 1.

## Discussion and conclusions

In this scoping review, 18 characteristic criteria were identified, of which 9 particularly differentiate the SIH from other approaches. The findings indicate that although the SIH concept is in construction, it is advancing down a path of recognition in the region and defining its role as an important field of study under which there are new studies that provide key components and methodological guidance reported in SIH case studies.

Delimitating criteria that characterise SIH has been an objective for SIHI Global as well as for institutions and their members interested in the subject. This search revealed various efforts from different hubs in the world, including that in Latin America. Kpokiri *et al* 18 provided a checklist that defined 17 items important for describing SIHI, of which 13 items are compatible with those identified in this review, while 4 are not directly related.

SIH, understood as a participatory process that seeks to operationalise the SDGs into concrete actions and achievements in the communities,^3^ conveys the importance of aligning solutions to local health problems with public health policies and, especially, with the SDGs. This alignment can ensure that community health is compatible with global health perspectives. Although SIH is an approach and conceptual category under construction, it can be adopted as a benchmark for health initiatives that aim at major commitments such as social development and universal health coverage.

It was possible to identify the four most similar approaches to SIH: (1) Ecohealth (94%), (2) SI (3) Sustainable Territories (83%) (4) Psychosocial Approach (78%). The 13 most consistent criteria of the sample were also recognised (table 3). These findings reveal that SIH requires the inclusion of additional theoretical and methodological constructs that, in a complementary manner, serve as a reference and foundation for innovative health solutions that benefit communities and transform local health problems. In this light, the relevance of the SIH approach is that it draws from knowledge obtained by different types of initiatives, leading to the promotion of better health outcomes and impacting more communities.

**Table 3:** Most consistent criteria found

Criteria		Approaches					
		Ecohealth	Social innovation	Sustainable and sanitary territories	Psychosocial approach	Sustainable development and health promotion	Environmental health
1	Focus on health promotion	✓	✓	✓	✓	✓	✓
2	Improves access to healthcare services	✓	✓	✓	✓	✓	✓
3	Local level development with socioecological perspectives on health	✓	✓	✓	✓	✓	✓
4	Active participation of civil society	✓	✓	✓	✓	✓	✓
5	Looks for sustainability	✓	✓	✓	✓	✓	✓
6	Promotes interdisciplinarity and intersectorality	✓	✓	✓	✓	✓	✓
7	Horizontalisation of power relations with a gender perspective	✓	✓	✓	✓	✓	✓
8	Aligns with the model of the social determinants of health.	✓	✓	✓	✓	✓	✓
9	Addresses specific health needs and has an additional impact on other	✓		✓	✓	✓	✓
10	Promotes human progress in social environments	✓	✓	✓	*	✓	✓
11	Emphasises scientific evidence	✓		✓	✓	✓	✓
12	Promotes resourcefulness	✓	✓	✓	✓	✓	
13	Implements health education approach strategies	✓		✓	✓	✓	

*Conclusive information was not found.

In summary, despite the dispersion of the approaches shown in this analysis, it is possible to suggest that SIH articulates different approaches and that it is suitable to be considered as a reference approach to correctly identify and typify health initiatives. In addition, the identification of these 18 criteria that describe and characterise it, makes it possible to advance towards the conceptual and methodological delimitation of SIH as a process or an approach. Based on these criteria, an initiative, proposal or project developed in community settings could be recognised in terms of its proximity to SIH according to which criteria it meets.

Many health interventions being implemented in communities that could be qualified as SIH, are possibly not being recognised as such, nor being studied; perhaps due to the lack of knowledge and delimitation of SIH.^3^ Not knowing what works adequately and what does not, is a limitation and creates evidence gaps to fill in the construction of this approach. The opportunity to know about, share, discuss and disseminate the lessons learnt from experiences that seek to solve local health problems are fundamental exercises when promoting the scalability and transferability of successful initiatives and may be missed in this sense.^19^

Authors such as Finkelman^20^ identified that some of the key criteria for SIH can also be considered as criteria that guarantee successful social transformations in health. Some criteria are related to promoting empowerment, social value and community ownership, leadership through strong participation of various stakeholders,^21 22^ multisectorality,^23 24^ contributing to the advancement of the SDGs or MDGs,^15 25^ contributing to social development being adaptable and promoting results with less investment than conventional alternatives. Successful initiatives must consider community mobilisation,^24 26^ community engagement and articulation across sectors.

To conclude, this contrasting exercise shows that Latin American health initiatives are working towards the improvement of the health conditions of different human groups majorly at a local level. The different approaches are betting on initiatives that include some degree of innovation, improve access to health services and recognise in one way or another, policies for development. Finally, it can be said that identifying approaches that are conceptually and methodologically more distant from SIH, sets conceptual boundaries to avoid confusing SIH for technological innovations in health, innovation in health and other kinds of interventions that promote health and prevent disease in community settings.

## Supplementary Material

Reviewer comments

## Data Availability

Data sharing not applicable as no datasets generated and/or analysed for this study. This article is made freely available for use in accordance with BMJ’s website terms and conditions for the duration of the COVID-19 pandemic or until otherwise determined by BMJ. You may use, download and print the article for any lawful, non-commercial purpose (including text and data mining) provided that all copyright notices and trademarks are retained.
