# Supplementary material for: Identification of social innovation in health criteria in Latin America
Source: BMJ Open. 2022 Jun 9;12(6):e063205. doi: 10.1136/bmjopen-2022-063205 (PMC9240894; doi:10.1136/bmjopen-2022-063205)
Supplement: Reviewer comments [file bmjopen-2022-063205.reviewer_comments.pdf]

PEER REVIEW HISTORY

BMJ Open publishes all reviews undertaken for accepted manuscripts. Reviewers are asked to complete a checklist review form (**http://bmjopen.bmj.com/site/about/resources/checklist.pdf**) and are provided with free text boxes to elaborate on their assessment. These free text comments are reproduced below.

ARTICLE DETAILS

**TITLE (PROVISIONAL)**	Identification of social innovation in health criteria in Latin America
**AUTHORS**	Duque-Paz, Luisa Fernanda; Castro-Arroyave, Diana

This article was not externally reviewed at BMJ Open. Protocols that have been independently assessed prior to submission to BMJ Open are usually fast-tracked to publication on the grounds that further substantial changes will not be possible. This independent assessment will usually be external, independent review for both a substantial grant award from a non-commercial or government funder and ethics approval.
